# Osteocalcin ameliorates cognitive dysfunctions in a mouse model of Alzheimer’s Disease by reducing amyloid β burden and upregulating glycolysis in neuroglia

**DOI:** 10.1038/s41420-023-01343-y

**Published:** 2023-02-06

**Authors:** Chang Shan, Deng Zhang, Dong-ni Ma, Yan-fang Hou, Qian-qian Zhuang, Yan-ling Gong, Li-hao Sun, Hong-yan Zhao, Bei Tao, Yu-ying Yang, Sheng-tian Li, Jian-min Liu

**Affiliations:** 1 Department of Endocrine and Metabolic Diseases, Shanghai Institute of Endocrine and Metabolic Diseases, Rui-jin Hospital, Shanghai Jiao Tong University School of Medicine, 200025 Shanghai, China; 2 Shanghai National Clinical Research Center for metabolic Diseases, Key Laboratory for Endocrine and Metabolic Diseases of the National Health Commission of the PR China, Shanghai National Center for Translational Medicine, Rui-jin Hospital, Shanghai Jiao Tong University School of Medicine, 200025 Shanghai, China; 3 Bio-X Institutes, Key laboratory for the Genetics of Developmental and Neuropsychiatric Disorders (Ministry of Education), Shanghai Key Laboratory of Psychotic Disorders, Brain Science and Technology Research Center, Shanghai Jiao Tong University, 200240 Shanghai, China

**Keywords:** Alzheimer's disease, Alzheimer's disease, Astrocyte, Microglia

## Abstract

Alzheimer’s disease (AD) is the most common neurodegenerative disease characterized by the accumulation of amyloid β peptides (Aβ) and impaired glucose metabolism in the brain. Osteocalcin (OCN), an osteoblast-derived protein, has been shown to modulate brain functions but whether it has any effect on AD is undetermined. In this study, daily intraperitoneal injection of OCN for 4 weeks ameliorated the anxiety-like behaviors and cognitive dysfunctions in the APP/PS1 transgenic AD mice model, as shown in the increased entries into the central area in open field test, the increased time and entries into open arms in elevated plus maze test, the increased time spent in the light chamber in light-dark transition test, as well as the reduced escape latency and the increased preference for target quadrant in Morris water maze test. Aβ burden in the hippocampus and cortex of AD mice was ameliorated by OCN. Besides, OCN improved the neural network function of the brain, mainly in the enhanced power of high gamma band in the medial prefrontal cortex of AD mice. The proliferation of astrocytes in the hippocampus in AD mice was also inhibited by OCN as demonstrated by immunofluorescence. Furthermore, OCN enhanced glycolysis in astrocytes and microglia, as evidenced by elevated glucose consumption, lactate production, and increased extracellular acidification rate. Such an effect was abolished when the receptor of OCN – Gpr158 was knockdown in astrocytes. Our study revealed OCN as a novel therapeutic factor for AD potentially through reducing Aβ burden and upregulation of glycolysis in neuroglia.

## Introduction

Alzheimer’s disease (AD) is the most common neurodegenerative disease which causes a progressive decline of cognition, and for which there is no cure [1]. Accumulation of Aβ in cognition-related brain regions is the primary factor driving AD pathogenesis [2]. However, Aβ-targeted therapeutics for AD have not yet proven to benefit significantly in clinical practice, which drives researches to explore new mechanisms and treatments.

Neuroglial cells, which mainly consist of astrocytes and microglia in central nervous system (CNS), function as the supporter for neurons by maintaining the microenvironment in CNS [3]. Specifically, astrocytes absorb glucose from blood and produce lactate via aerobic glycolysis. Astrocyte-derived lactate is taken by neurons and converted to pyruvate and further undergoes oxidative phosphorylation (OXPHOS) to fuel neuronal activity [4]. Alteration of brain aerobic glycolysis is often observed early in the course of AD [5, 6]. In cultures of human fetal astrocytes, inhibition of main enzymatic regulator of glycolysis results in increased accumulation of Aβ within and around astrocytes and greater vulnerability of these cells to Aβ toxicity [7]. On the other hand, increasing aerobic glycolysis ameliorates the Aβ-induced activation of astrocytes and improves the spatial cognition in AD mice [5, 8]. Thus, targeting glycolysis in neuroglia, especially in astrocytes, may be a promising therapy for AD treatment. However, there are currently few these kinds of drugs in the market. Thus, safe and effective molecules are needed to be discovered.

Osteocalcin (OCN), a polypeptide secreted exclusively by osteoblasts, exerts multiple endocrine functions through its metabolically active undercarboxylated form (ucOCN) [9]. Besides its regulations of glucose metabolism, lipid metabolism, muscle power, and male fertility [10–14], an intriguing role of OCN in CNS is being revealed. OCN can pass through the blood–brain barrier (BBB) and modulating the monoamine neurotransmitters and favoring learning and memory of mice. Moreover, maternal OCN can cross the placenta to regulate the development of fetal hippocampus and maintain postnatal neurogenesis [15]. Giving aged mice with exogenous OCN or OCN-containing young mice plasma could improve the cognitive functions of the aged mice [16]. Further studies identified that G-protein-coupled receptor 158 (Gpr158) is the receptor in CNS that OCN binds and combats age-related hippocampal-dependent cognitive impairments [16]. The above evidence suggests that OCN has a protective effect on age-related cognitive decline. However, whether OCN can play a role in improving cognition in a disease state like AD is still unknown.

Based on the regulatory role of OCN in the hippocampus and its protection of age-related cognitive dysfunctions, our study aimed to explore whether OCN can ameliorate the cognitive dysfunctions in a mouse model of AD and investigate the potential mechanisms, trying to expand the endocrine functions of OCN and providing a new dimension for the treatment of AD.

## Results

### OCN ameliorated the anxiety-like behaviors and cognitive dysfunctions in AD mice

**Fig. 1:**
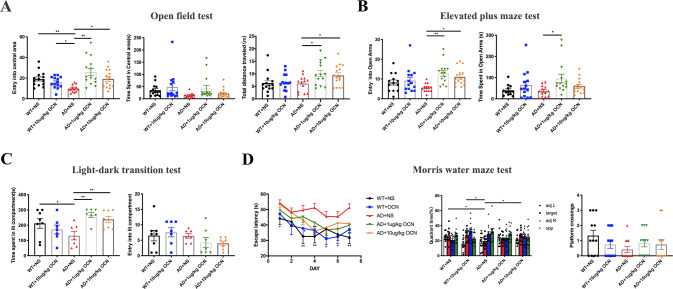
OCN ameliorated the anxiety-like behaviors and cognitive dysfunctions in APP/PS1 transgenic AD mouse model. In the Open Field Test (**A**), OCN significantly increased the entries into the central area and total distance traveled in AD mice, especially in 1 μg/kg OCN group (*n* = 13–15 per group). In the Elevated Plus Maze Test (**B**), OCN increased the time and entries that AD mice entered into open arms, especially in the 1 μg/kg OCN group (*n* = 13–15 per group). In the Light-Dark Transition Test (**C**), OCN significantly increased the time of AD mice spent in the light chamber (*n* = 7–8 per group). In the Morris Water Maze Test (**D**), AD mice exhibited increased escape latency during the training stage, which was decreased by OCN treatment. In the probe test, 10 μg/kg OCN significantly increased the preference for target quadrant in AD mice (*n* = 12 per group). All data represent the means ± SEM. A *P*-value < 0.05 was considered to be statistically significant by one-way ANOVA followed by post hoc comparisons using LSD’s test or Kruskal–wallis test followed by post hoc comparisons using Dunn’s test for multiple groups’ comparisons. *P < 0.05, **P < 0.01.

In view of the fact that OCN can cross the BBB, we tried to identify whether OCN could ameliorate the anxiety-related behaviors and learning and memory impairments of AD mice by daily intraperitoneal injection of 1 μg/kg or 10 μg/kg OCN for 4 weeks. Within 5-min free exploration in the OFT, entries into the central area in AD mice were significantly less than that in WT mice (*P* = 0.004, Fig. 1A), while OCN significantly increased the entries of AD mice into the central area, especially in 1 μg/kg OCN group (*P* = 0.006 and 0.011 respectively, Fig. 1A). Although there was no statistical difference (*P* > 0.05), time spent in the central area of open field had a decreased trend in AD mice (*P* = 0.078. Figure 1A), and OCN treatment had an increased trend in the time spent in the central area of AD mice, especially in the 1 μg/kg OCN group (Fig. 1A). Besides, OCN significantly increased total distance traveled in AD mice (*P* = 0.011 in 1 μg/kg OCN group and 0.035 in 10 μg/kg OCN group respectively, Fig. 1A).

In the EPMT, AD mice tended to have decreased entries into the open arms compared with WT mice, while OCN significantly increased the entries that AD mice entered into open arms, especially in the 1 μg/kg OCN group (*P* = 0.002 and 0.032 respectively, Fig. 1B). Besides, 1 μg/kg OCN significantly increased the time that AD mice spent in open arms (Fig. 1B). In the LDTT, time spent in the light chamber of AD mice was significantly less than that of WT mice (*P* = 0.033, Fig. 1C). 1 μg/kg (*P* = 0.001) and 10 μg/kg (*P* = 0.006) OCN treatment significantly increased the time of AD mice spent in the light chamber (Fig. 1C). There was no significant difference in the entries into the light chamber among groups (*P* = 0.571, Fig. 1C). In the above behavioral experiments, there was no significant difference between WT + 10 μg/kg OCN and WT + NS group (*P* > 0.05). Data of the above experiments were presented in Table S1.

In MWMT, AD mice exhibited significantly increased escape latency during the training stage, which was decreased by OCN treatment (Fig. 1D). In the probe test, the preference for target quadrant was compromised in AD mice compared with WT mice (*P* = 0.019 compared with WT + NS group, *P* = 0.012 compared with WT + 10 μg/kg OCN group, Fig. 1D), and 10 μg/kg OCN treatment significantly increased the preference for target quadrant in AD mice (*P* = 0.014, Fig. 1D). In addition, although there was no significant difference, there was an increasing trend in the crossings of the previous platform after OCN treatment in AD mice (*P* > 0.05, Fig. 1D). Data of the MWMT were presented in Table S2.

### OCN reduced the Aβ burden in the hippocampus and cortex of AD mice

**Fig. 2:**
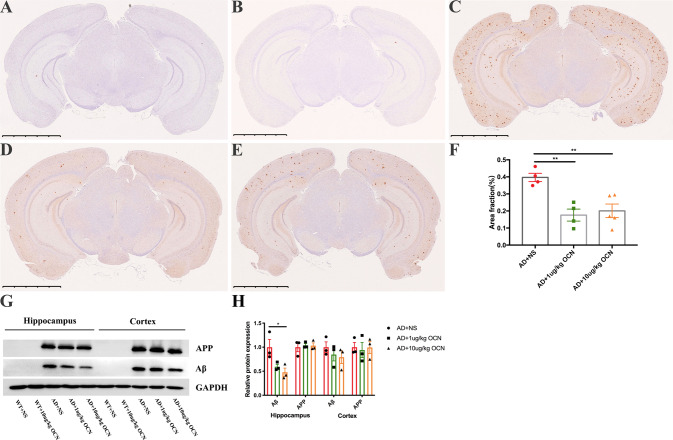
OCN reduced the Aβ burden in the hippocampus and cortex of AD mice. **A**–**E** Represent immunostaining showing Aβ burden in the hippocampus and cortex. (**A**) WT + NS; (**B**) WT + 10 μg/kg OCN; (**C**) AD + NS; (**D**) AD + 1 μg/kg OCN; (**E**) AD + 10 μg/kg OCN. **F** Quantitation of Aβ-positive area fraction (*n* = 4–5 per group). **G** Represent western blot showing Aβ and APP protein levels in hippocampus and cortex. **H** The quantitation of Aβ and APP protein levels in hippocampus and cortex (*n* = 3 per group). All data represent the means ± SEM. A *P*-value < 0.05 was considered to be statistically significant by one-way ANOVA followed by post hoc comparisons using Turkey’s test for multiple groups’ comparisons. *P < 0.05, **P < 0.01.

In order to explore the reasons for behavioral alterations in AD mice after intraperitoneal injection of OCN, we further detected whether there were pathological changes of Aβ burden in the hippocampus and cortex of mice. As shown in Fig. 2, obvious amyloid plaques were observed in the hippocampus and cortex of AD mice (Fig. 2C), but not in WT mice (Fig. 2A, B). 1 μg/kg and 10 μg/kg OCN could significantly reduce the burden of amyloid plaques in AD mice (*P* = 0.004 and *P* = 0.007 respectively, Fig. 2D, E). The quantitative data were shown in Fig. 2F. Similar results were found in western blot, that is, no expression of APP and Aβ protein were observed in the hippocampus and cortex of WT mice, while there were high expressions in AD mice (Fig. 2G). OCN had a dose-dependent effect in reducing Aβ in the hippocampus and cortex of AD mice, but only 10 μg/kg OCN achieved a statistically significant reduction in Aβ in the hippocampus (*P* = 0.035, Fig. 2G, H). No significant reduction of APP in the hippocampus and cortex was observed by OCN treatment (Fig. 2G, H).

### OCN altered the power of LFP in the mPFC of AD mice

**Fig. 3:**
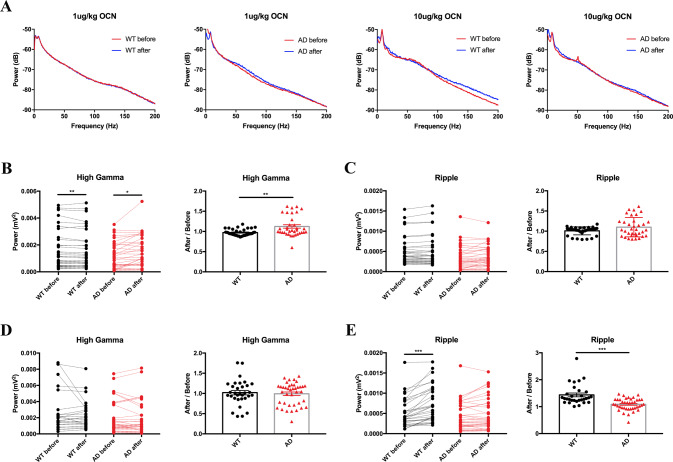
OCN altered the LFP power in the mPFC. **A** 1 μg/kg OCN altered the LFP power at 0–200 Hz bands in AD mice, while 10 μg/kg OCN altered the LFP power in WT mice. **B** 1 μg/kg OCN increased the LFP power at High gamma (55–90 Hz) band both in WT and AD mice. Oscillations of High gamma band were higher in AD mice than WT mice after 1 μg/kg OCN treatment. **C** 1 μg/kg OCN did not affect the LFP power at Ripple band (100–200 Hz). **D** 10 μg/kg OCN did not affect the LFP power at High gamma band. **E** 10 μg/kg OCN increased the LFP power at Ripple band in WT mice. Oscillations of Ripple band were higher in WT mice than AD mice after 10 μg/kg OCN treatment. *n* = 5 per group. A *P*-value < 0.05 was considered to be statistically significant by paired *t* test when comparing the difference between before and after OCN treatment, unpaired *t* test when comparing the difference between WT group and AD group. **P < 0.01, ***P < 0.001.

LFP recordings represent summed synaptic activity across networks of neurons and often exhibit voltage oscillations in mammals [17]. Particular oscillation frequencies have been found to be associated with behavioral function and neural connectivity [17]. To determine whether OCN plays a role in the synaptic activity in the mPFC of AD mice, we examined how LFP power altered after 1 and 10 μg/kg OCN intraperitoneal injection. As is shown, when analyzing the LFP power at 0–200 Hz bands in AD mice, 1 μg/kg OCN but not 10 μg/kg OCN altered the LFP power (Fig. 3A). However, when analyzing in WT mice, 10 μg/kg OCN but not 1 μg/kg OCN altered the LFP power (Fig. 3A). Especially, 1 μg/kg OCN significantly increased the LFP power at High gamma band both in WT and AD mice (Fig. 3B), but not at Ripple (Fig. 3C), Theta (Fig. S1A) and Beta bands (Fig. S1B). While 10 μg/kg OCN significantly increased the LFP power at Ripple band (Fig. 3E), but not at High gamma (Fig. 3D), Theta (Fig. S1C) and Beta bands (Fig. S1D). When comparing the oscillations of LFP power after OCN injection between WT and AD mice, power of high gamma band oscillation showed significant difference after 1 μg/kg OCN injection (Fig. 3B), while power of Ripple band oscillation showed significant difference after 10 μg/kg OCN injection (Fig. 3E). No significant differences were found in other bands oscillations after OCN treatment (Fig. S1). Data of the LFP at in vivo multichannel electrophysiological recording were presented in Table S3 and Table S4.

### OCN inhibited the proliferation of astrocytes in the hippocampus of AD mice

**Fig. 4:**
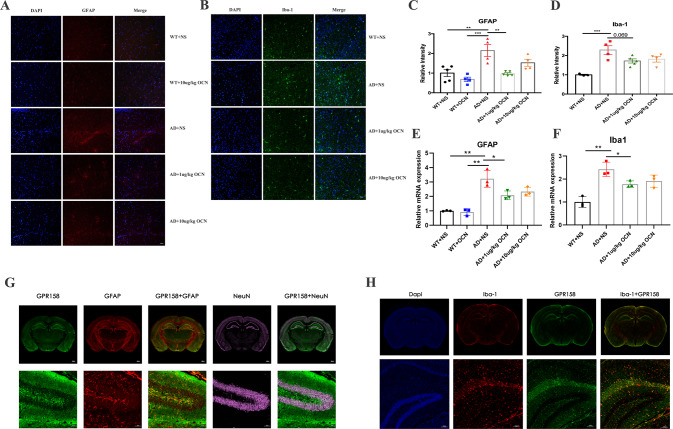
OCN inhibited the proliferation of astrocytes in the hippocampus of AD mice, and Gpr158 is mainly co-localized with astrocytes in hippocampus. **A** Represent immunofluorescence showing GFAP in the hippocampus. **B** Represent immunofluorescence showing Iba-1 in the hippocampus. **C** Quantitation of relative GFAP-positive intensity in the hippocampus (*n* = 4–5 per group). **D** the quantitation of relative Iba-1-positive intensity in the hippocampus (*n* = 3–5 per group). **E** Relative mRNA expression of GFAP in the hippocampus (*n* = 3 per group). **F** Relative mRNA expression of Iba-1 in the hippocampus (*n* = 3 per group). **G** Gpr158 is co-localized with astrocytes in hippocampus. **H** Gpr158 is not mainly co-localized with microglia in hippocampus. All data represent the means ± SEM. A *P*-value < 0.05 was considered to be statistically significant by one-way ANOVA followed by post hoc comparisons using Turkey’s test for multiple groups’ comparisons. **P < 0.01, ***P < 0.001.

Mounting evidence have shown that activation of glial cells, including astrocytes and microglia, not only plays an important role in neuroinflammation but also is a key player in mediating Aβ generation, neuronal loss and brain glucose dysregulation involved in neurodegeneration [18–20]. Thus, we stained GFAP and Iba1-positive cells in the hippocampus of mice. A significant proliferation of astrocytes was observed in AD mice (*P* = 0.002, Fig. 4A, C), while intraperitoneal injection of 1 μg/kg OCN could significantly inhibit this phenomenon in AD mice (*P* = 0.002, Fig. 4A, C). As for microglia, results from the Iba-1 staining showed that there was an upregulation of Iba1-positive microglia numbers in the hippocampus of AD mice (*P* < 0.001, Fig. 4B, D), while 1 μg/kg OCN had a borderline effect in reducing this upregulation of Iba1-positive microglia numbers (*P* = 0.069, Fig. 4B, D). Similar results regarding the expressions of GFAP and Iba-1 were observed in qPCR (Fig. 4E, F) and western blot (Fig. S2).

Studies have shown that Gpr158 mediates the signal of OCN in the CNS [16, 21]. Thus, we co-stained Gpr158 and NeuN (a marker of neurons) with GFAP (marker of astrocytes) and Iba-1 (a marker of microglia) in the hippocampus of mice. As shown in Fig. 4G, H, Gpr158 was mainly co-localized with astrocytes rather than microglia and neurons.

### OCN-enhanced glycolysis in astrocytes and microglia

**Fig. 5:**
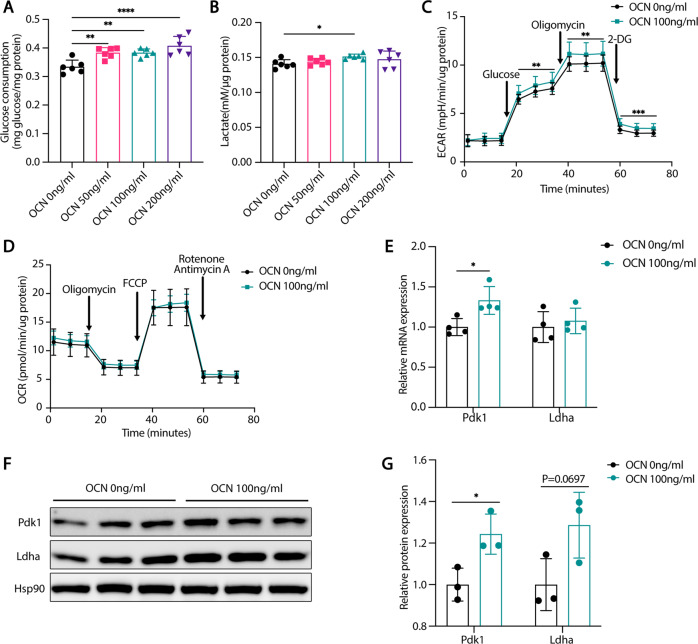
OCN enhanced glycolysis in astrocytes. **A** Glucose consumption (normalized to total protein) of C8D1A cells treated with vehicle or different concentrations of OCN. **B** Lactate production (normalized to total protein) of C8D1A cells treated with vehicle or different concentrations of OCN. **C**, **D** Oxygen consumption rate (OCR) and extracellular acidification rate (ECAR) of C8D1A cells treated with vehicle or 100 ng/ml of OCN. Results are normalized to total protein. **E** qPCR analysis of mRNA levels of glycolytic enzymes of C8D1A cells treated with vehicle or 100 ng/ml of OCN. **F**, **G** Protein levels of glycolytic enzymes of C8D1A cells treated with vehicle or 100 ng/ml of OCN. (Data are presented as mean ± SD).

Metabolic dysregulation is one of the major pathological changes in AD [8, 22]. Aβ activates the astrocytes and impairs its energy metabolism mainly characterized by a decrease in glycolysis [23]. Here we found that 50 ng/ml to 200 ng/ml OCN significantly increased the glucose consumption of C8D1A cell (a cell line of astrocytes) (Fig. 5A). Furthermore, lactate concentrations in culture medium, representing the glycolytic activity of cultured cells, were elevated in OCN-treated C8D1A cells (Fig. 5B). As the maximum lactate concentration was reached when 100 ng/ml OCN was administrated, we used this concentration for further experiments. Using Seahorse real-time metabolic analyzer, we found that OCN promoted maximum glycolytic capacity in C8D1A cells (Fig. 5C). Meanwhile, OXPHOS was unaffected by OCN (Fig. 5D). Pdk1 is a key enzyme increasing glycolytic flux and Ldha is the key enzyme in lactate production [24, 25]. OCN upregulated Pdk1 at both mRNA and protein levels while upregulated Ldha protein level of a marginal significance (*P* = 0.070) (Fig. 5E–G).

**Fig. 6:**
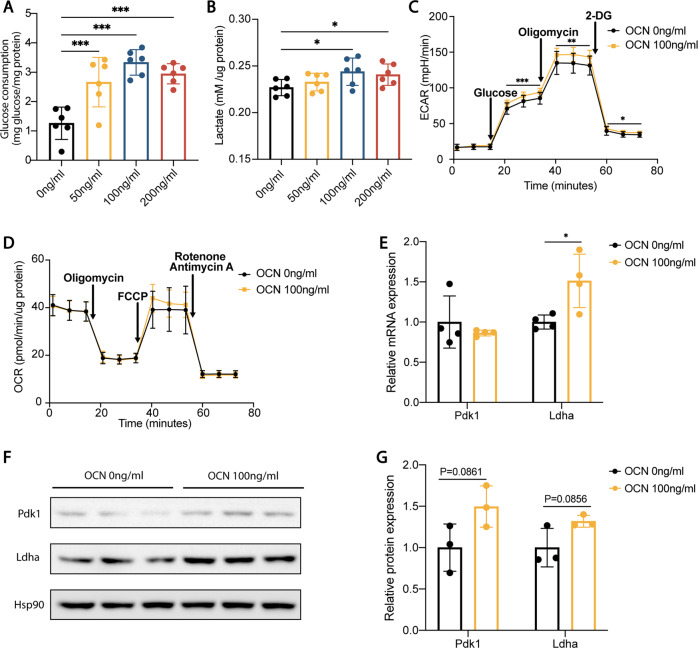
OCN enhanced glycolysis in microglia. **A** Glucose consumption (normalized to total protein) of BV2 cells treated with vehicle or different concentrations of OCN. **B** Lactate production (normalized to total protein) of BV2 cells treated with vehicle or different concentrations of OCN. **C**, **D** Oxygen consumption rate (OCR) and extracellular acidification rate (ECAR) of BV2 cells treated with vehicle or 100 ng/ml of OCN. Results are normalized to total protein. **E** qPCR analysis of mRNA levels of glycolytic enzymes of BV2 cells treated with vehicle or 100 ng/ml of OCN. **F**, **G** Protein levels of glycolytic enzymes of BV2 cells treated with vehicle or 100 ng/ml of OCN. (Data are presented as mean ± SD).

Recent study evidenced that chronic treatment with Aβ disrupts glycolysis in microglia, while stimulating glycolysis restores the immunological functions of microglia [26]. In our present study, OCN promoted glucose consumption as well as lactate production in BV2 cells (a cell line of microglia) (Fig. 6A, B). The glycolytic stress test showed that OCN increased maximum glycolytic capacity in BV2 cells (Fig. 6C). Mitochondria stress test showed that OCN did not change OXPHOS in BV2 cells (Fig. 6D). The mRNA levels of Ldha were upregulated while that of Pdk1 were not affected (Fig. 6E). Protein levels of Pdk1 and Ldha were elevated of a marginal significance (*P* = 0.086 and *P* = 0.086, respectively) (Fig. 6F, G).

Apart from astrocyte and microglia, we also tested the effect of OCN on neurons. OCN increased glucose consumption in HT22 cells (Fig. S3A) while lactate production (Fig. S3B), ECAR in glycolysis stress test (Fig. S3C), and OCR in mitochondria stress test (Fig. S3D) were not significantly changed by OCN treatment.

### Ablation of GPR158 blocked the effect of OCN on glycolysis in astrocytes

**Fig. 7:**
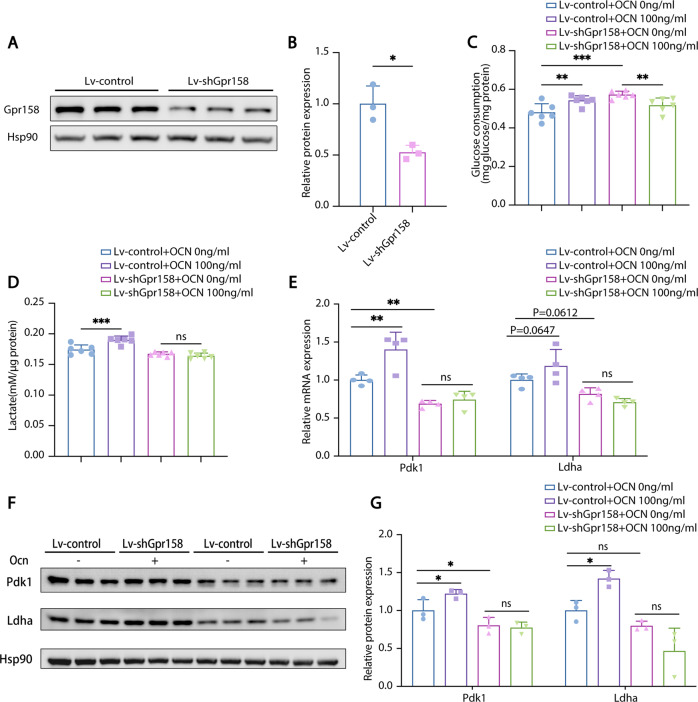
Ablation of GPR158 blocked the effect of OCN on glycolysis in astrocytes. **A**, **B** Protein levels of Gpr158 in control and Gpr158 knockdown C8D1A cells. **C** Glucose consumption (normalized to total protein) of control and Gpr158 knockdown C8D1A cells treated with vehicle or 100 ng/ml of OCN. **D** Lactate production (normalized to total protein) of control and Gpr158 knockdown C8D1A cells treated with vehicle or 100 ng/ml of OCN. **E** qPCR analysis of mRNA levels of glycolytic enzymes of control and Gpr158 knockdown C8D1A cells treated with vehicle or 100 ng/ml of OCN. **F**, **G** Protein levels of glycolytic enzymes of control and Gpr158 knockdown C8D1A cells treated with vehicle or 100 ng/ml of OCN. (Data are presented as mean ± SD).

It is reported that Gpr158 is the receptor responsible for the cognition-regulatory function of OCN [16]. As described above, we found that Gpr158 is also expressed in astrocytes and microglia, however, it is unknown if OCN improves glycolysis through Gpr158. We interfered Gpr158 expression by virus-delivered shRNA in C8D1A cells and the Gpr158 protein was downregulated by 56% (Fig. 7A, B). The positive effect of OCN on glucose consumption and lactate production was abolished in Gpr158 knockdown cells (Fig. 7C, D). Interestingly, we found that Gpr158 knockdown cells consumed more glucose than control cells while the lactate production trend to decrease (Fig. 7C, D). Regarding this phenomenon, we hypothesized that OXPHOS is elevated in Gpr158 knockdown cells. To test this hypothesis, we determined the OCR during mitochondria stress test and results showed that OCR was significantly higher in Gpr158 knockdown cells (Fig. S4). Furthermore, Pdk1 were downregulated in Gpr158 knockdown cells and both mRNA and protein levels of Pdk1 and Ldha did not respond to the OCN treatment in Gpr158 knockdown cells (Fig. 7E–G).

## Discussion

This study found that OCN could ameliorate anxiety-like behaviors and cognitive dysfunctions, reduce the Aβ burden, and inhibit the proliferation of astrocytes in the hippocampus in a mouse model of AD. Besides, OCN altered the power of LFP in the mPFC of AD mice, especially increased the LFP power at high gamma band. In vitro study showed that OCN increased aerobic glycolysis in astrocytes and microglia. Ablation of Gpr158 in astrocytes abolished the effect of OCN on glycolysis.

The close interaction between bone and other organ, including CNS is a research hotspot in recent years [27]. Bone is emerging as a multi-faceted endocrine organ by secreting several osteokines including OCN [28, 29]. A previous study has demonstrated the role of OCN in combating age-related cognitive impairments [16], but whether it can play a neuroprotective role in AD-dependent memory loss has not been discovered. We found that OCN significantly increased the times of APP/PS1 mice entering the center of OFT and the open arm of EMPT. Besides, OCN significantly increased the time of APP/PS1 mice spent in the light chamber of LDTT. In addition, OCN increased the spatial learning and memory ability of APP/PS1 mice in the MWMT. These findings provide evidence for the neuroprotective effects of OCN in ameliorating the anxiety-like behaviors and memory loss in AD mice, but how OCN plays this protective role is still unclear.

Accumulation of Aβ in the brain putatively leads to neurodegeneration with cognitive impairments and increases the likelihood of progression from mild cognitive impairment to dementia [30, 31]. Our study found that OCN treatment for 4 weeks could reduce 49.4–55.7% of the Aβ burden in the hippocampus and cortex of AD mice, which is in line with other studies that 40–60% reduction of Aβ could exhibit a cognition improvement in AD mice [32, 33]. APP is the precursor of the Aβ peptides and APP mutations affect Aβ cleavage and aggregation [34]. In our study, Aβ in hippocampus and cortex of AD mice was significantly reduced by OCN treatment but the high expression of APP was not affected, which suggested that OCN might play a role in promoting the clearance of Aβ, thus reducing the Aβ burden.

Neural oscillations of particular frequency are critical mechanisms enabling dynamic coupling of corresponding brain regions and coordinated activity during normal brain functioning [35, 36]. We found that OCN increased the LFP power at high gamma band in the mPFC. This echoed our results of improved cognition of AD mice, because increased high gamma band oscillations occur during the transient brain states that are associated with attention, stimulus recognition and are prominent in the cortical-hippocampal network, which is responsible for cognitive processing and a variety of memory tasks in rats, monkeys, and humans [37–39]. More importantly, high gamma rhythms may facilitate direct transmission from the medial entorhinal cortex that transmits ongoing spatial information. Increased high gamma rhythms in the hippocampus promote encoding of novel object-place pairings in rats [40]. Our results demonstrated an increase of LFP power at high gamma band in WT and AD mice after OCN treatment and this effect seems to be more significant in AD mice, which might be the physiological base of improved memory.

Despite the above results indicating neuronal function is improved in AD mice model by OCN, the underlying mechanism is unknown. Studies utilizing ^18^F-deoxyglucose positron emission tomography (^18^FDG-PET) demonstrate that there is a decrease of glucose uptake in cerebral regions vulnerable to AD pathology [41]. Subsequent studies suggest that AD is a central metabolic disorder characterized by an early and progressive cerebral glucose dysregulation [42]. Especially, changes in aerobic glycolysis prevail in the early phase of AD [43] and compared to neurons, glias in CNS are more relied on aerobic glycolysis [44]. Glial cells have been recently implicated in the pathogenesis AD. During the development of AD, various cerebral lesions including Aβ exposure can cause reactive astrocytes to proliferate [45]. In our study, proliferation of astrocytes and microglia was observed in hippocampus of AD mice, which was inhibited by OCN. As the hub of neurovascular unit, astrocytes are the main energy suppliers for neurons [4]. Disruption of astrocyte metabolic homeostasis impairs astrocyte-neuron metabolic coupling, impairs Aβ clearance and leads to neurodegeneration over time [46]. On the other hand, accumulated Aβ, in turn, impairs glycolysis which leads to a vicious cycle finally results in neuronal damage [23]. Our study demonstrated that OCN favors aerobic glycolysis in astrocytes as evidenced by elevated glucose consumption and lactate production. It is well established that Pdk1 enhances glycolysis and lactate production in many cells types [47, 48]. We found that OCN upregulated Pdk1 at both mRNA level and protein level in astrocytes. However, more research is needed to investigate whether Pdk1 is indispensable in the regulation of glycolysis by OCN. These results indicate that OCN might break the above-mentioned vicious cycle in astrocytes thus leading to less Aβ accumulation and improved neuronal function. Similar to our results, Zheng et al. revealed that glucagon-like peptide-1 (GLP-1) improve cognition of AD mice through upregulating glycolysis of astrocytes [8]. These novel findings highlighted glycolysis as a promising target for AD treatment and OCN, an endogenous molecule, as a novel regulator of glycolysis of astrocytes.

After investigating the role of OCN in astrocyte, we further studied the impact of OCN on microglia. A recent study has shown that chronic Aβ treatment leads to a decrease in the production of lactate and a defect in glycolytic metabolism in microglia. Boosting glycolytic metabolism with interferon-γ reduced Aβ plaques, neuronal losses, and cognitive impairment in an AD mouse model [26]. In our study, we showed that OCN favors glycolysis in microglia, revealing a new role of OCN in ameliorating cognition defects in AD by regulating glucose metabolism of glias.

Our data showed that OCN improves glycolysis both in astrocytes and microglia but not in neurons. It is understandable because neurons predominantly undergo oxidative phosphorylation using astrocytes-derived lactate to produce pyruvate which is further catabolized through OXPHOS to generate ATP [4, 44, 49]. Thus, according to our results, the neuroprotective function of OCN is closely related with its effect through favoring aerobic glycolysis of glias.

Since the receptor of OCN in the CNS is Gpr158 [16, 21], to further confirm whether Gpr158 mediates the effect of OCN on astrocytes, we co-stained Gpr158 with GFAP and found that Gpr158 is expressed in astrocytes in the hippocampus. Glucose consumption and lactate production did not respond to OCN treatment in Gpr158 knockdown astrocytes. Besides, we found an interesting phenomenon that glucose consumption, but not lactate production, was higher in Gpr158 knockdown cells than in control cells. Further study demonstrated that this was due to elevated OXPHOS flux in Gpr158 knockdown cells, indicating there are other effects on glucose metabolism that Gpr158 mediates besides the effect of OCN on glycolysis. Indeed, studies have found that the Gpr158 form a complex with regulator of G protein signaling 7 (RGS7) to regulate controlling stress-induced changes in neuronal excitability [50]. More studies are needed to further investigate the OCN-independent function of Gpr158 in astrocytes.

There are some limitations related to this study. Firstly, whether the aerobic glycolysis is downregulated in cognition-related cerebral regions of AD mice and whether OCN can alter this phenomenon were not explored in our study. However, other studies have clarified that AD mice displayed localized decreases in glucose uptake in the amygdala, cortex, and hippocampus [26], which provided evidence to support our study. Moreover, down-regulation of Gpr158 was not conducted in vivo to confirm whether OCN plays a beneficial role in AD via combination with this receptor. Further studies are warranted to identify these questions.

In summary, our study demonstrated that OCN could exert a neuroprotective effect in ameliorating the cognitive deficits in AD mice by enhancing aerobic glycolysis in astrocytes and microglia. Besides, the regulatory roles of OCN in the high gamma band oscillation in the mPFC may attribute to its neuroprotective effects in AD. Our study provides more evidence for a better understanding of the crosstalk between bone and CNS, and expands the neuroprotective roles of OCN in AD, suggesting that OCN or its derived analogs may be explored as a potential therapeutic approach in neurodegenerative diseases like AD.

## Materials and methods

### Animals

All animal experiments in this study were approved by the ethics committee of Shanghai Jiao Tong University (SJTU) and conducted according to the National Institutes of Health Guidelines for the Care and Use of Laboratory Animals (NIH Publications No. 8023, revised 1978). 6-month-old B6C3-Tg (APPswePSEN1dE9) /Nju male mice were purchased from Nanjing University-Nanjing Institute of Biomedicine (Nanjing, China) and used as the AD mice model in this study, which express both the mutant human presenilin 1 (PS1-dE9) and human mouse amyloid preprotein (APPswe) fusion. Wild-type (WT) male littermates were used as controls. All the mice were housed under a 12 h light/dark cycle at the Laboratory Animal Center of SJTU with free access to food and water.

Age and weight-matched WT littermates and APP/PS1 male mice were randomly assigned to five groups: the WT + normal saline (NS) group, the WT + 10 μg/kg OCN group, the AD + NS group, the AD + 1 μg/kg OCN group and the AD + 10 μg/kg OCN group (*N* = 15 per group). 1 mg ucOCN (H-6552, Bachem, Bubendorf, Switzerland) was dissolved in NS to a concentration of 0.2 or 2 μg/ml, and all the mice received daily intraperitoneal injection of 5 ml/kg ucOCN of different concentrations or 5 ml/kg NS for 4 weeks and then were subjected to the behavioral tests. The duration of OCN’s administration was chosen based on a previous study regarding OCN’s protective role of age-related cognitive dysfunction [16]. Five mice died before the behavioral tests. After the behavioral tests, mice were sacrificed for western blot and immunostaining.

### Behavioral tests

#### Open field test (OFT)

OFT is a commonly used method to evaluate the locomotor activity. It is also a popular procedure in animal phycology, which indicates anxiety with the activity in the central area of the open field [51]. OFT was performed as previously described [52, 53]. Briefly, mice were placed into the center of the OFT apparatus (a white 27.5 × 27.5 cm rectangular chamber with an open top) and allowed to explore freely for 5 min under low illumination. All activities inside the chamber were monitored by a video camera and tracked using the ANY-maze automated video system (Version 4.115; Stoelting Co., Wood Dale, IL, USA) [54]. Time spent in the central area, entries into the central area of OFT chamber and total distance traveled were used to reflect the anxiety state of the mice. One mouse died after OFT.

#### Elevated plus maze test (EPMT)

EPMT is also routinely used to study the anxiety-related behaviors of mice [55]. It takes advantage of the innate aversion of rodents to open fields [56]. The plus maze apparatus, which consists of two open arms and two closed arms, was placed directly below the camera at the height of 60 cm from the ground. Mice were placed into the center of the plus maze chamber facing one closed arm and allowed to explore freely for 5 min. All activities inside the chamber were monitored by a video camera and tracked using the ANY-maze automated video system (Version 4.115; Stoelting Co., Wood Dale, IL, USA). Time spent in the open arms and entries into the open arms were analyzed.

#### Light-dark transition test (LDTT)

LDTT exploits the approach-avoidance conflict of mice between the aversion to bright fields and the novelty-induced exploratory behaviors [57]. The apparatus is composed of an open chamber with white floor (30 cm * 60 cm) equipped with infrared light sensors and one equivalent dark chamber with black walls and lid, which is connected by an opening (13 cm * 5 cm) for mice to shuttle freely. The apparatus was placed directly below the camera and the light chamber was illuminated by two 40 W light bulbs. Mice were released in the center of the light chamber and allowed to explore freely for 5 min. Time spent in the light chamber and entries into the light chamber were recorded to measure the anxiety-like behavior of mice.

#### Morris water maze test (MWMT)

MWMT is a classical method to test the spatial learning and memory ability of rodents [58]. Five mice died before MWMT. Mice were transported to the testing room in their home cages the day before the first training day. The maze consists of a 150-cm diameter circular pool with a 10-cm round platform, which was placed 1 cm below the surface of the water during the training stage. The pool was filled with water opaque with edible titanium dioxide and the water temperature was kept around 22 °C. High contrast spatial cues were pasted on the walls of the testing room at 3 o’clock, 6 o’clock, 9 o’clock and 12 o’clock of the pool. When being tested, the animal could not see the experimenter [59].

The whole course of this test was eight days, of which Days 1st–7th were the training stage while Day 8th was the probe test stage. Each mouse was trained four times a day during the training stage with the automatically randomized chosen starting position. To begin testing, the mouse was lifted by the base of the tail and gently placed into the water, facing the edge of the pool. Mice that failed to find the platform within 60 sec were guided to the platform, and all the mice were allowed to stay on the platform for 30 sec to remember the platform position. Escape latency was recorded for each mouse and each day, and the average time of four trials was used for statistical analysis. On Day 8th for probe test, each mouse was tested only once. The platform was removed, and the mice were released from one starting direction, allowed to explore freely in the water maze for 60 sec. Times each mouse passed through the previous platform position and the percent of time spent in each quadrant were recorded automatically by the software [59]. Four mice that did not swim in the pool were excluded from the analysis.

### In vivo multichannel electrophysiological recording

In vivo multichannel electrophysiological recording was used to investigate the influence of ucOCN on neurons in medial prefrontal cortex (mPFC). An 8-channel microelectrode array was fabricated. The array was arranged in 1 × 8 configuration: 33μm diameter nickel-chromium wires with formvar insulation, 0.25 mm inter-electrode spacing and impedance 100–250 kΩ. In our study, microelectrodes were inserted into 5 APP/PS1 mice and 5 WT littermates. Implantation of the electrode was described as previously [60, 61]. Briefly, mice were deeply anesthetized with 1% pentobarbital sodium and then fixed onto the brain stereotaxic apparatus. The electrode was slowly advanced into mPFC (AP: 1.9 mm, ML: 0.5 mm, DV: 1.7 mm), with reference and ground electrodes fixed in the skull above the cerebellum. Subsequently, the electrodes were tightly secured to the skull with dental cement. Mice were allowed to recover for a week before recording. Using the signal acquisition software equipped with Bourne’s NeuroStudio system, the high-frequency spike signal and the low-frequency signal could be simultaneously displayed and the original unfiltered signal could be recorded. Recording was started when the stable and high signal-to-noise signal was observed. Signals were recorded for 30 min before intraperitoneal injection of ucOCN (1 or 10 μg/kg) and 60 min after ucOCN injection, respectively. Local field potential (LFP) data were analyzed with a MATLAB software (Version R2020a, MathWorks Inc., MA, USA). Sub-band analyses were conducted at Theta (4–4.5 Hz), Beta (13–25 Hz), High gamma (55–90 Hz) and Ripple (100–200 Hz) bands.

### Western Blot

Brain tissues or cell pellets were lysed in ice-cold RIPA buffer (R0278, Sigma, St. Louis, MO) supplemented with protease inhibitor cocktail (4693159001, Roche, Mannkin, Germany) and phosphatase inhibitor cocktail (4906845001, Roche, Mannkin, Germany), followed by centrifugation at 4 °C to collect the supernatant. Total protein concentration was quantified with BCA protein assay reagents (23227, Thermo Fisher, Fair Lawn, NJ, USA). Equal amounts of total proteins were separated with 6–10% sodium dodecyl sulfate‐polyacrylamide gel electrophoresis (SDS-PAGE) and then transferred onto PVDF membranes. Membranes were incubated with 5% non-fat milk in 0.1% TBST for 2 h at room temperature, then incubated with primary antibody overnight at 4 °C. After incubated with HRP-conjugated secondary antibodies for 2 h at room temperature, blots were developed with an electrochemiluminescence (ECL) reagent (34577, Thermo Fisher, Fair Lawn, NJ, USA). Primary antibodies used in this study were as follows: mouse anti-Aβ 1–16 (1: 1000 dilution, 803001, BioLegend), mouse anti-Gapdh (1: 5000 dilution, ab8245, Abcam), rabbit anti Pdk-1 (1: 500 dilution, 3820 S, Cell Signaling Technology), rabbit anti-Ldha (1: 1000 dilution, 2012S, Cell Signaling Technology), mouse anti-Hsp90 (1: 1000 dilution, sc-13119, Santa Cruz Biotechnology), rabbit anti-Gpr158 (1: 500 dilution, SAB4502509, Sigma-Aldrich), mouse anti-GFAP (1: 1000 dilution, MAB360, Sigma-Aldrich), and rabbit anti-Iba1 (1: 500 dilution, ab178847, Abcam).

### Total RNA extraction and qPCR analysis

Total RNA was extracted from tissues or cultured cells using Eastep® Super Total RNA Extraction Kit (Promega, Beijing, China) according to the manufacturer’s instructions. The RNA concentration and absorbance ratio at 260/280 nm of all samples were checked using a NanoDrop ND2000 spectrophotometer (Thermo Scientific, Waltham, MA, USA). Reverse transcription was performed using the PrimeScript™ Reverse TranscriptMasterMix (TaKaRa Bio, Otsu, Japan). qPCR was conducted using the QuantStudio™Dx Real-Time PCR Instrument (Applied Biosystems, Foster City, CA, USA). The comparative computed tomography (CT) method was used to evaluate the relative messenger RNA (mRNA) levels and relative gene expression which was normalized to 36b4. Primer sequences are listed in Table S5.

### Immunohistochemistry

After the behavioral tests, 5 mice per group were deeply anesthetized with 1% pentobarbital sodium (0.8 ml/kg) and intracardially perfusion-fixed with 4% paraformaldehyde (PFA) in 0.1 M phosphate buffer (pH 7.4). Brains were completely dissected, post-fixed in 4% PFA, dehydrated in 20–30 (w/v) % sucrose, and subsequently embedded in paraffin. Later, brains were sectioned at a thickness of 4 um and every fifth section containing the hippocampus was collected. The brain sections were inactivated by 3% H2O2, blocked with 5% goat serum, and then incubated with primary antibody against Aβ 1–16 (6E10) (1: 400 dilution, mouse anti-human, 803001, Biolegend, San Diego, CA, USA) overnight at 4 °C. Anti-Aβ 1–16 antibody (6E10) recognizes the N terminus of human Aβ and binds to the monomer, parenchymal plaques, and cerebral amyloid angiopathy [62]. On the second day, the sections were incubated with a biotin-conjugated goat anti-mouse IgG secondary antibody, and the following staining was made using the avidin-biotin complex (ABC) system (PK-6100, Vectastain Elite, Vector labs, Burlingame, CA) and nickel-enhanced diaminobenzidine (DAB) incubation (SK-4100, Vector labs, Burlingame, CA). Images were obtained using a microscope (Olympus IX70, Japan) and fractions of Aβ 1–16-positive area in the hippocampus and cortex were measured using ImageJ 1.49 v (National Institutes of Health, USA).

### Immunofluorescence

4–5 mice per group were sacrificed for immunofluorescence. Brains were embedded in O.C.T. compound and frozen sections of 30 um thickness were cut on a cryostat (CM1860; Leica Microsystems). The brain sections were permeabilized with 0.3% Triton X-100, blocked with 5% goat serum, and then incubated with rabbit anti-Iba-1 (1: 1000 dilution, 019-19741, Wako) or mouse anti-GFAP (1: 2000 dilution, 8152 S, Cell Signaling Technology) overnight at 4 °C. After being washed three times, they were incubated for 2 h at room temperature in the dark with appropriate secondary antibody conjugated with FITC or PE and DAPI (P0131, Beyotime, China). The sections were mounted with fluorescent mounting medium. Images were obtained using a fluorescence microscope (AMG EVOS FL Microscope, USA).

Tyramide signal amplification (TSA) method was used for co-staining GPR158 and other biomarkers. For co-staining GPR158 and NeuN, GFAP, and DAPI, brain sections were incubated with rabbit anti-GPR158 antibody (1:200 dilution, G307, AssayBioTech) overnight at 4 °C. On the next day, slices were balanced at room temperature for 1 h. After washed with PBS for 3 times, secondary antibody (1:2000 dilution, ab205718, Abcam) were added and incubated at 37 °C for 30 min. After washed with PBS for 3 times, try-488 (Bry-try488, Runnerbio, China) were added and incubated at room temperature for 30 min. Similarly, the second antibody rabbit anti-GFAP antibody (1: 2000 dilution, 8152 S, Cell Signaling Technology) plus try-cy3 (Bry-trycy3, Runnerbio, China) and the third antibody Rabbit anti-NeuN (1: 3000 dilution, ab177487, Abcam) plus try-cy5 (Bry-trycy5, Runnerbio, China) were added. For co-staining GPR158 and Iba-1, the first primary antibody rabbit anti-Iba-1 antibody (1: 1000 dilution, 019-19741, Wako) plus try-488 and the second primary antibody rabbit anti-GPR158 antibody (1:200 dilution, G307, AssayBioTech) plus try-cy3 were added as previously described. Slices were mounted with antifade mounting medium with DAPI (P0131, Beyotime, China).

### Cell culture

Murine hippocampal neuronal HT22 cells, astrocyte type I clone cells (C8-D1A) and microglial cells (BV2) were used in this study to explore the role of ucOCN in regulating aerobic glycolysis in different cell types. Cell lines were cultured with the complete medium, composed of Dulbecco’s Modified Eagle’s Medium (DMEM, 11965092, Gibco, Logan, UT) supplemented with 10% fetal bovine serum (10099141 C, Gibco, Logan, UT) and 1% penicillin/streptomycin (15140122, Gibco, Logan, UT), and the medium was replaced every 2–3 days. Cell lines were maintained in a 5% CO2 humidified atmosphere at 37 °C and treated for 2 h with different concentrations of ucOCN (50 ng/ml, 100 ng/ml, and 200 ng/ml).

### Glucose consumption assays and lactate measurements

Glucose consumption and lactate levels were measured according to the manufacturer’s protocols. Cells were cultured in 96-well plates and treated with or without ucOCN for 2 h. For glucose consumption measurement, fresh media and the media cultured cells for 2 h were assayed with Glucose (HK) Assay Kit (GAHK20, Sigma-Aldrich, St. Louis, MO, USA) under the instructions and was corrected for total protein content in each well. For lactate measurements, the cultured medium was measured using the L-lactate assay kit (1200011002, Eton Biosciences, Union, NJ, USA) from Eton Biosciences.

### Measurement of oxygen consumption rate and extracellular acidification rate

For oxygen consumption rate (OCR) and extracellular acidification rate (ECAR) measurement, cells were seeded in an XF V3 microplate (Seahorse Bioscience, Billerica, ME, USA) coated with poly-L-lysine, and measured using a Seahorse XFe 96 extracellular flux analyzer (Seahorse Biosciences, Agilent Technologies, USA) following the manufacturer’s instructions. For OCR, the culture medium of the cells was changed to Seahorse XF base medium with 25 mM D-glucose, 2 mM sodium pyruvate, and 2mM L-Glutamine (LG), and incubated at 37 °C in a non-CO2 incubator (Seahorse Bioscience) for 1 h. Respiratory inhibitors (103015-100, Seahorse Bioscience, North Billerica, Massachusetts, USA) were loaded into the injection port to reach the final concentration of 1 μM oligomycin, 2 μM carbonyl cyanide-p-trifluoromethoxyphenylhydrazone (FCCP), 0.5 μM antimycin A, and 0.5 μM rotenone to detect the uncoupled respiration, maximal respiration, and non-mitochondrial respiration, respectively.

For ECAR, the culture medium of the cells was changed to Seahorse XF base medium with 2 mM LG and incubated at 37 °C in a non-CO2 incubator (Seahorse Bioscience) for 1 h. Reagents were loaded into the injection port in sequence to reach the final concentration of 10 mM glucose, 2 μM oligomycin, and 0.1 M 2-Deoxy-D-glucose (2-DG). The final OCR and ECAR results were normalized to total protein content.

### Statistical analysis

Statistical analyses were conducted with SPSS 24.0 (IBM, Armonk, NY, USA), GraphPad Prism 7 (GraphPad Software Inc., San Diego, CA, USA) or Sigma Plot 14.0 (Systat Software Inc., San Jose, CA, USA). One-way ANOVA or Kruskal–wallis test followed by post hoc comparisons was used to assess the statistical significance of differences among multiple groups. Paired *t* test or Wilcoxon test was used to compare the difference before and after OCN treatment, and unpaired *t* - test or Mann-Whitney test was used to compare the difference between WT and AD mice in in vivo multichannel electrophysiological recording. Data were expressed as means ± SEM in vivo and means ± SD in vitro. A *P*-value < 0.05 was considered to be statistically significant.

## Supplementary information

SUPPLEMENTARY FIGURES

SUPPLEMENTARY TABLES

Original full length western blots

## Data Availability

Some or all datasets generated during and/or analyzed during the current study are not publicly available but are available from the corresponding author on reasonable request.
