# Supplementary material for: Osteocalcin ameliorates cognitive dysfunctions in a mouse model of Alzheimer’s Disease by reducing amyloid β burden and upregulating glycolysis in neuroglia
Source: Cell Death Discov. 2023 Feb 6;9:46. doi: 10.1038/s41420-023-01343-y (PMC9902399; doi:10.1038/s41420-023-01343-y)
Supplement: Supplementary file 1 — SUPPLEMENTARY FIGURES [file 41420_2023_1343_MOESM1_ESM.docx]

**SUPPLEMENTARY FIGURE LEGENDS**

**Figure S1.** OCN did not alter the LFP power in the mPFC at Theta and Beta band. **Figure S2.** Protein levels of GFAP and Iba-1 in the hippocampus. **Figure S3.** OCN didn’t change glycolysis in neurons. **Figure S4.** Oxygen consumption rate (OCR) of control and Gpr158 knockdown C8D1A cells treated with vehicle or 100 ng/ml of OCN.


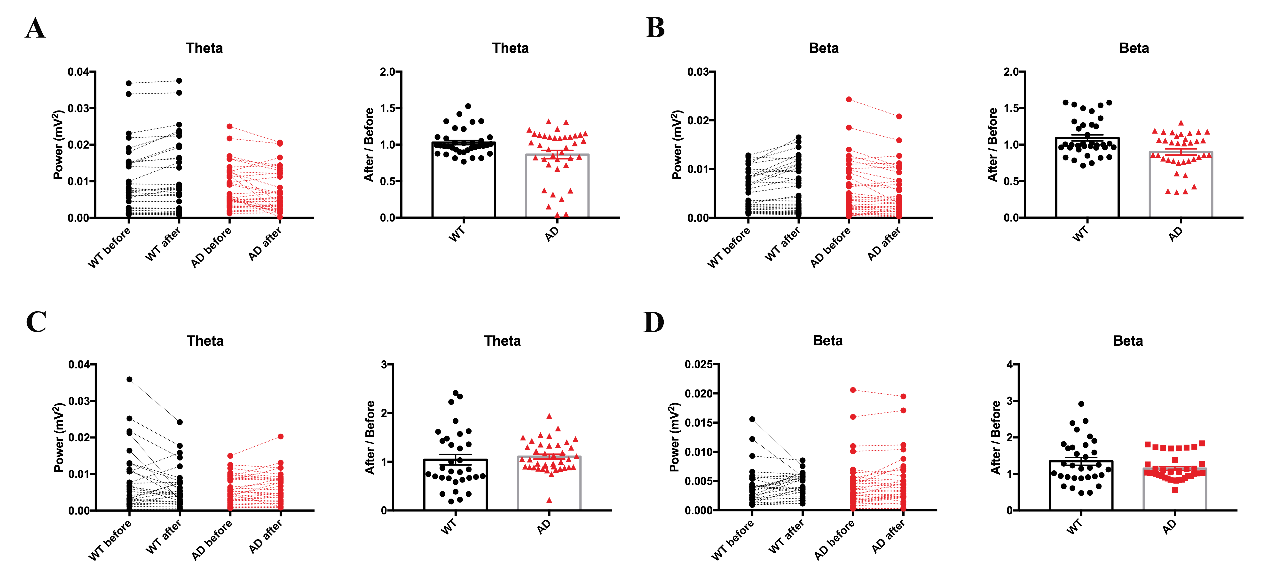


**Figure S1.** OCN did not alter the LFP power in the mPFC at Theta and Beta band. A, 1 ug/kg OCN did not alter the LFP power at Theta band (4 - 4.5 Hz) in WT and AD mice. B, 1 ug/kg OCN did not alter the LFP power at Beta band (13 - 25 Hz) in WT and AD mice. C, 10 ug/kg OCN did not alter the LFP power at Theta band in WT and AD mice. D, 10 ug/kg OCN did not alter the LFP power at Beta band in WT and AD mice. n = 5 per group. A P-value < 0.05 was considered to be statistically significant by paired t-test when comparing the difference between before and after OCN treatment, unpaired t-test when comparing the difference between WT group and AD group.


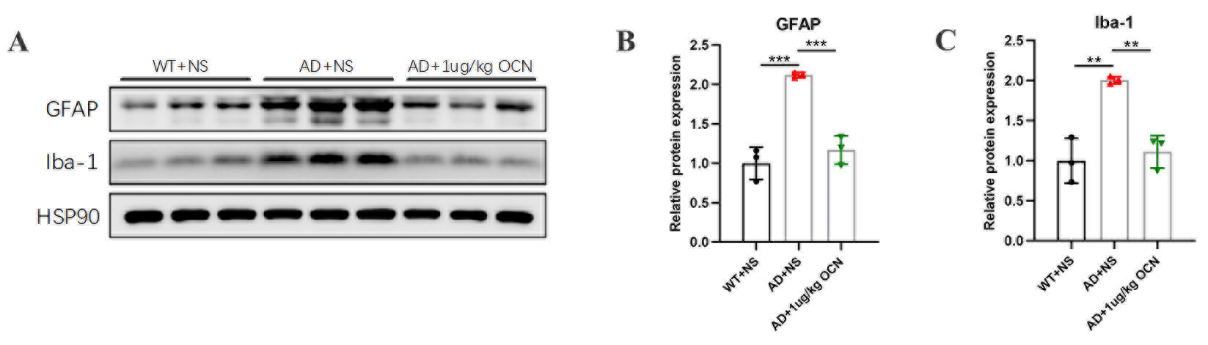


**Figure S2.** Protein levels of GFAP and Iba-1 in the hippocampus. A, the expression of GFAP and Iba-1 in the hippocampus in western blot. B, relative protein expression of GFAP in the hippocampus (n = 3 per group). C, relative protein expression of Iba-1 in the hippocampus (n = 3 per group).


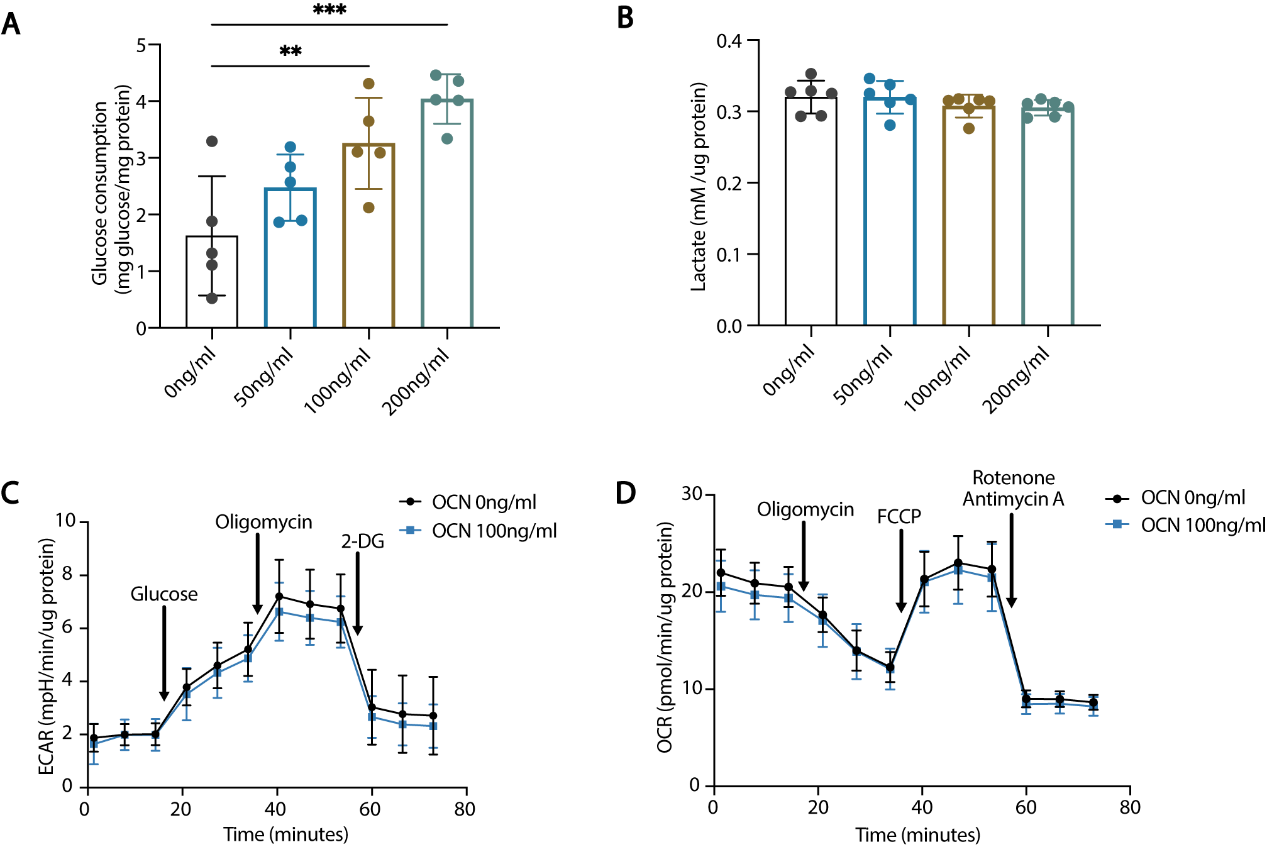


**Figure S3.** OCN didn’t change glycolysis in neurons. A, Glucose consumption (normalized to total protein) of HT22 cells treated with vehicle or different concentrations of OCN. B, Lactate production (normalized to total protein) of HT22 cells treated with vehicle or different concentrations of OCN. C-D, Oxygen consumption rate (OCR) and extracellular acidification rate (ECAR) of HT22 cells treated with vehicle or 100 ng/ml of OCN. Results are normalized to total protein. (Data are presented as mean ± SD).


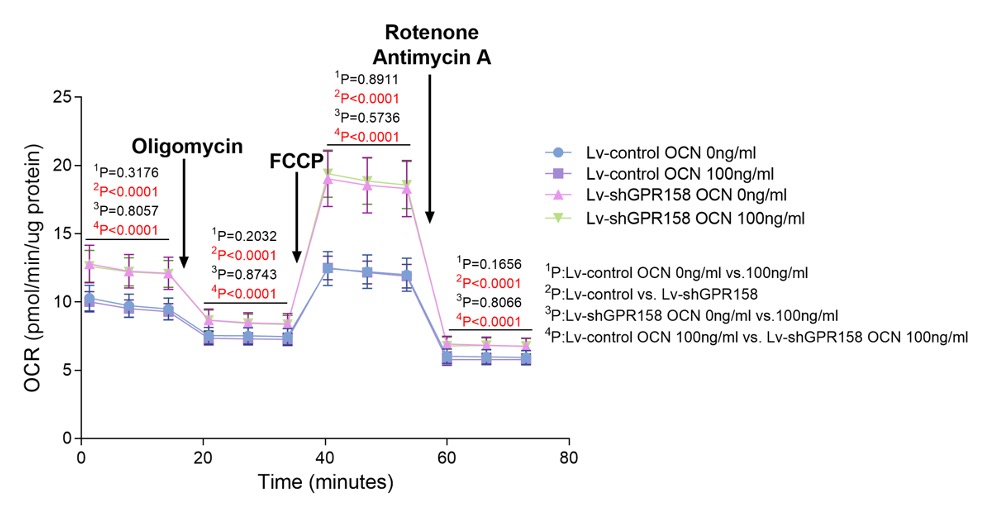


**Figure S4.** Oxygen consumption rate (OCR) of control and Gpr158 knockdown C8D1A cells treated with vehicle or 100 ng/ml of OCN. Results are normalized to total protein. (Data are presented as mean ± SD).
