# Supplementary material for: Osteocalcin ameliorates cognitive dysfunctions in a mouse model of Alzheimer’s Disease by reducing amyloid β burden and upregulating glycolysis in neuroglia
Source: Cell Death Discov. 2023 Feb 6;9:46. doi: 10.1038/s41420-023-01343-y (PMC9902399; doi:10.1038/s41420-023-01343-y)
Supplement: Supplementary file 2 — SUPPLEMENTARY TABLES [file 41420_2023_1343_MOESM2_ESM.docx]

**SUPPLEMENTARY TABLE LEGENDS**

**Supplemental Table 1.** Data of open field test, elevated plus maze test and light-dark transition test in WT and AD mice. **Supplemental Table 2.** Data of Morris Water Maze Test in WT and AD mice. **Supplemental Table 3.** Data of LFP before and after intraperitoneal 1 ug/kg OCN injection by in vivo multichannel electrophysiological recording. **Supplemental Table 4.** Data of LFP before and after intraperitoneal 10 ug/kg OCN injection by in vivo multichannel electrophysiological recording. **Supplemental Table 5.** Primers used for real-time PCR.

**Supplemental Table 1.** Data of open field test, elevated plus maze test and light-dark transition test in WT and AD mice.

| **Behavioral Tests** | **WT + NS** | **WT+10ug/kg OCN** | **AD + NS** | **AD+1ug/kg OCN** | **AD+10ug/kg OCN** |
| --- | --- | --- | --- | --- | --- |
| **Open Field Test** | N = 14 | N = 14 | N = 13 | N = 14 | N = 15 |
| Time spent in Central area (s) | 35.143 ± 6.480 | 47.950 ± 15.783 | 14.546 ± 2.579 | 42.150 ± 13.331 | 21.753 ± 5.267 |
| Entry into Center | 19.21 ± 1.953^**^ | 15.79 ± 1.502^*^ | 9.85 ± 0.953^#^ | 25.86 ± 3.561^**^ | 19.20 ± 2.256^*^ |
| Total distance traveled (m) | 6.331 ± 1.071 | 6.678 ± 0.893 | 6.143 ± 0.866 | 10.104 ± 1.287^*^ | 9.361 ± 1.071^*^ |
| **Elevated Plus Maze Test** | N = 13 | N = 14 | N = 13 | N = 14 | N = 15 |
| Time spent in Open arm (s) | 39.746 ± 6.929 | 65.429 ± 17.913 | 36.885 ± 7.031 | 95.564 ± 20.680^*^ | 59.547 ± 8.995 |
| Entry into open arm | 8.23 ± 1.277 | 9.21 ± 1.681 | 5.62 ± 0.594 | 12.93 ± 1.484^**^ | 11.07 ± 1.123^*^ |
| **Light-dark Transition Test** | N = 8 | N = 7 | N = 7 | N = 7 | N = 8 |
| Time spent in Lit Compartment (s) | 211.750 ± 31.590^*^ | 171.729 ± 29.374 | 130.914 ± 27.336^#^ | 267.200 ± 17.181^**^ | 237.825 ± 19.925^**^ |
| Entry into Lit Compartment | 6.50 ± 1.701 | 7.57 ± 1.478 | 6.43 ± 0.751 | 5.43 ± 2.626 | 4.00 ± 0.655 |

Note: * significantly compare to the AD + NS group. # significantly compare to the WT + NS group. * P<0.05, ** P<0.01; # P<0.05.

**Supplemental Table 2.** Data of Morris Water Maze Test in WT and AD mice.

| **Morris Water Maze Test** | **WT+NS**  **(N = 12)** | **WT+10ug/kg OCN (N = 12)** | **AD+NS (N = 12)** | **AD+1ug/kg OCN (N = 12)** | **AD+10ug/kg OCN (N = 12)** |
| --- | --- | --- | --- | --- | --- |
| **Training (Escape latency)** |  |  |  |  |  |
| First day | 44.162 ± 3.842^*^ | 47.225 ± 2.958 | 54.206 ± 2.038^#^ | 49.726 ± 3.030 | 53.187 ± 1.971 |
| Second day | 42.153 ± 2.770 | 39.446 ± 5.726 | 48.214 ± 2.781 | 44.171 ± 3.839 | 47.916 ± 3.144 |
| Third day | 32.581 ± 4.584^**^ | 37.829 ± 4.613^*^ | 49.589 ± 3.175^##^ | 45.302 ± 3.516 | 43.222 ± 2.858 |
| Fourth day | 32.586 ± 4.187^**^ | 36.498 ± 2.741^*^ | 51.131 ± 2.954^##^ | 41.511 ± 3.771 | 36.021 ± 3.624^*^ |
| Fifth day | 37.274 ± 3.846^**^ | 31.096 ± 3.877 | 45.452 ± 3.345^##^ | 37.037 ± 4.968 | 38.346 ± 3.665 |
| Sixth day | 33.922 ± 3.761^*^ | 32.427 ± 4.358^*^ | 45.355 ± 3.366^#^ | 37.376 ± 4.498 | 41.291 ± 4.133 |
| Seventh day | 31.958 ± 3.971^**^ | 37.104 ± 5.843^**^ | 51.219 ± 2.474^##^ | 39.893 ± 3.526^**^ | 40.979 ± 3.648 |
| **Probe Test** |  |  |  |  |  |
| Time percentage in target quadrant (%) | 26.891 ± 2.663^*^ | 27.411 ± 2.243^*^ | 19.462 ± 1.991^#^ | 22.135 ± 1.732 | 27.306 ± 2.141^*^ |
| Platform Crossings | 1.33 ± 0.355 | 0.75 ± 0.250 | 0.42 ± 0.193 | 0.83 ± 0.241 | 0.75 ± 0.329 |

Note: * significantly compare to the AD group. # significantly compare to the WT group. *P<0.05, ** P<0.01; #P<0.05.

**Supplemental Table 3.** Data of LFP before and after intraperitoneal 1 ug/kg OCN injection by in vivo multichannel electrophysiological recording

| **Band** | **WT before** | **WT after** | **P** | **AD before** | **AD after** | **P** | **WT oscillations** | **AD oscillations** | **P** |
| --- | --- | --- | --- | --- | --- | --- | --- | --- | --- |
| **Theta** (4-4.5 Hz) | 0.00655 (0.00135-0.0149) | 0.00648 (0.00125-0.0164) | 0.229 | 0.00561 (0.00391-0.0126) | 0.00537 (0.00235-0.0121) | 0.087 | 0.985 (0.905-1.098) | 0.975 (0.725-1.120) | 0.241 |
| **Beta** (13-25 Hz) | 0.00334 (0.00132-0.00839) | 0.00423 (0.00133-0.0109) | 0.187 | 0.00370 (0.00148-0.0097) | 0.00325 (0.00182-0.00763) | 0.151 | 0.999 (0.958-1.271) | 1.017 (0.776-1.148) | 0.140 |
| **High gamma** (55-90 Hz) | 0.000986 (0.000444-0.00230) | 0.000926 (0.000446-0.00220) | 0.002 | 0.000911 (0.000407-0.00195) | 0.00104 (0.000514-0.00218) | 0.013 | 0.947 (0.907-1.029) | 1.048 (0.975-1.474) | <0.001 |
| **Ripple** (100-200 Hz) | 0.000384 (0.000258-0.000600) | 0.000376 (0.000264-0.000607) | 0.090 | 0.000336 ± 0.0000481 | 0.000367 ± 0.0000440 | 0.0507 | 1.036 (0.987-1.067) | 1.051 (0.903-1.407) | 0.146 |

**Supplemental Table 4.** Data of LFP before and after intraperitoneal 10 ug/kg OCN injection by in vivo multichannel electrophysiological recording

| **Band** | **WT before** | **WT after** | **P** | **AD before** | **AD after** | **P** | **WT oscillations** | **AD oscillations** | **P** |
| --- | --- | --- | --- | --- | --- | --- | --- | --- | --- |
| **Theta** (4-4.5 Hz) | 0.00587 (0.00244-0.0112) | 0.00443 (0.00236-0.00773) | 0.079 | 0.00496 (0.00278-0.00881) | 0.00471 (0.00262-0.00850) | 0.632 | 0.823 (0.663-1.464) | 1.050 (0.886-1.340) | 0.106 |
| **Beta** (13-25 Hz) | 0.00360 (0.00183-0.00538) | 0.00393 (0.00291-0.00583) | 0.173 | 0.00303 (0.00155-0.00510) | 0.00343 (0.00228-0.00549) | 0.069 | 1.201 (0.912-1.775) | 1.035 (0.938-1.270) | 0.264 |
| **High gamma** (55-90 Hz) | 0.00179 (0.00108-0.00257) | 0.00170 (0.00113-0.00301) | 0.939 | 0.00120 (0.000639-0.00361) | 0.00115 (0.000571-0.00232) | 0.583 | 0.959 (0.869-1.189) | 1.106 (0.727-1.186) | 0.596 |
| **Ripple** (100-200 Hz) | 0.000406 (0.000280-0.000757) | 0.000677 (0.000389-0.00110) | <0.001 | 0.000306 (0.000181-0.000686) | 0.000304 (0.000200-0.000691) | 0.075 | 1.318 (1.232-1.687) | 1.090 (0.941-1.262) | <0.001 |

**Supplemental Table 5.** Primers used for real-time PCR

| gene | Primer sequences | |
| --- | --- | --- |
| GFAP | forward | CACGAACGAGTCCCTAGAGC |
|  | reverse | GGCTGGTTTCTCGGATCTGG |
| Iba-1 | forward | ATCAACAAGCAATTCCTCGATGA |
|  | reverse | CAGCATTCGCTTCAAGGACATA |
| Pdk1 | forward | GGACTTCGGGTCAGTGAATGC |
|  | reverse | TCCTGAGAAGATTGTCGGGGA |
| Ldha | forward | AGGCTCCCCAGAACAAGATT |
|  | reverse | TCTCGCCCTTGAGTTTGTCT |
| 36b4 | forward | GAAACTGCTGCCTCACATCCG |
|  | reverse | GCTGGCACAGTGACCTCACACG |
