# Supplementary material for: Osteocalcin ameliorates cognitive dysfunctions in a mouse model of Alzheimer’s Disease by reducing amyloid β burden and upregulating glycolysis in neuroglia
Source: Cell Death Discov. 2023 Feb 6;9:46. doi: 10.1038/s41420-023-01343-y (PMC9902399; doi:10.1038/s41420-023-01343-y)
Supplement: Supplementary file 3 — Original full length western blots [file 41420_2023_1343_MOESM3_ESM.docx]

**Figure 5F.** Original full length western blots

PDK1





LDHA





HSP90





**Figure 6F.** Original full length western blots

PDK1





LDHA





HSP90





**Figure 7A.** Original full length western blots

GPR158





HSP90





**Figure 7F.** Original full length western blots

PDK1





LDHA





HSP90





**Figure S2A.** Original full length western blots

GFAP





Iba1





HSP90
